# Isochorismate synthase is required for phylloquinone, but not salicylic acid biosynthesis in rice

**DOI:** 10.1007/s42994-024-00166-4

**Published:** 2024-05-24

**Authors:** Zengqian Wang, Guiqing Yang, Dandan Zhang, Guangxin Li, Jin-Long Qiu, Jie Wu

**Affiliations:** 1 College of Agriculture, Shanxi Agricultural University, Jinzhong, 030801 China; 2 State Key Laboratory of Plant Genomics, Institute of Microbiology, Chinese Academy of Sciences, Beijing, 100101 China

**Keywords:** Rice, Isochorismate synthase (ICS), Salicylic acid, Phylloquinone, CRISPR/Cas9

## Abstract

Salicylic acid (SA) is a phytohormone required for plant growth and defense signaling. There are two major SA biosynthesis pathways in plants: the isochorismate synthase (ICS) pathway and the phenylalanine ammonia-lyase (PAL) pathway. It has been demonstrated in several plant species, including the model plant *Arabidopsis*, that SA is derived predominantly from the ICS pathway. Here, we employed the CRISPR/Cas9 system to generate *ICS* knockout mutants in rice (*Oryza sativa* L.). The *Osics* mutants display severe growth defects, and are completely devoid of phylloquinone, an isochorismate-derived product. The growth defects of *Osics* can be rescued through exogenous application of 1,4-dihydroxy-2-naphthoic acid (NA), a precursor of phylloquinone. Remarkably, the basal SA levels are not altered in the *Osics* mutants. Our findings support a role of OsICS in the biosynthesis of phylloquinone, and imply that SA biosynthesis in rice may occur through an alternative route other than the ICS pathway.

**Supplementary Information:**

The online version contains supplementary material available at 10.1007/s42994-024-00166-4.

## Introduction

Salicylic acid (SA) not only functions as a pivotal phytohormone that mediates plant defense responses (Ding and Ding 2020), but also contributes to various aspects of plant growth and development, including photosynthesis, transpiration, seed germination, thermogenesis, drought resistance, and senescence (Khalvandi et al. 2021; Uzunova and Popova 2000; Vicente and Plasencia 2011; Vlot et al. 2009). Previous work acknowledges the existence of both the isochorismate synthase (ICS) and phenylalanine ammonia-lyase (PAL) pathways for SA biosynthesis in plants, in both cases SA originates from chorismate in the plastid (Lefevere et al. 2020). However, the exact SA biosynthesis process still remains largely unknown in lots of plant species. Dissecting the mechanism underlying plant SA biosynthesis will further our understanding of plant immunity and development, and may open avenues for developing novel strategies for crop improvement.

Genetic analysis of SA-deficient mutants has revealed that SA is predominantly synthesized through the ICS pathway in *Arabidopsis* and some other plant species, similar to the pathway described in *Pseudomonas* bacteria (Mercado-Blanco et al. 2001; Muller et al. 1996; Serino et al. 1995). The ICS pathway has been fully elucidated in *Arabidopsis*, in which chorismate is converted into isochorismate by ICS in plastid, and the resultant compound is exported to the cytosol to produce SA (Rekhter et al. 2019; Torrens-Spence et al. 2019). The ICS pathway accounts for over 90% of SA biosynthesis induced by pathogens or ultraviolet light in *Arabidopsis* (Garcion et al. 2008). The remaining 10% SA is believed to originate from the PAL pathway where chorismate undergoes a multistep enzymatic conversion into phenylalanine (Phe) and PAL converts Phe into *trans*-cinnamic acid, a precursor of SA (Yalpani et al. 1993). Interestingly, a recent isotopic tracing study in *Arabidopsi*s challenged the notion that SA is synthesized from Phe (Wu et al. 2023), suggesting the presence of a PAL-independent pathway.

The ICS pathway is also predominant in some other plants such as tomato (Uppalapati et al. 2007), maize (Djamei et al. 2011), and tobacco (Catinot et al. 2008). However, the relative contribution of the ICS pathway to SA biosynthesis varies among different plant species. In soybean (*Glycine max*), both the ICS and PAL pathways are crucial for pathogen-induced SA biosynthesis, as knocking down either pathway halts SA production and compromises pathogen resistance (Shine et al. 2016). In addition, the ICS pathway is also implicated in the biosynthesis of phylloquinone (known as vitamin K1), an essential component for electron transfer in photosystem I (PSI) (Furt et al. 2010; Yuan et al. 2009). In a barley (*Hordeum vulgare*) *ics* mutant, phylloquinone is deficient while the basal SA level remains unchanged, suggesting that SA in barley likely synthesized through an ICS-independent pathway (Qin et al. 2019).

While being one of the most important staple crops, rice (*Oryza sativa* L.) also serves as a widely-used model monocot. Notably, rice has relatively high basal SA levels, which surpass those observed in other model plants such as *Arabidopsis* and tobacco, as well as in other cereal species like wheat and maize (Klessig et al. 2016; Pál et al. 2014). In this study, we generated *Osics* mutants in rice using CRISPR/Cas9 system and found that these *Osics* plants are seedling lethal and deficient in phylloquinone. However, the SA levels in the *Osics* mutants remained unchanged, indicating that SA in rice is biosynthesized through an ICS-independent pathway.

## Results

### Characterization of ICS in rice

**Fig. 1:**
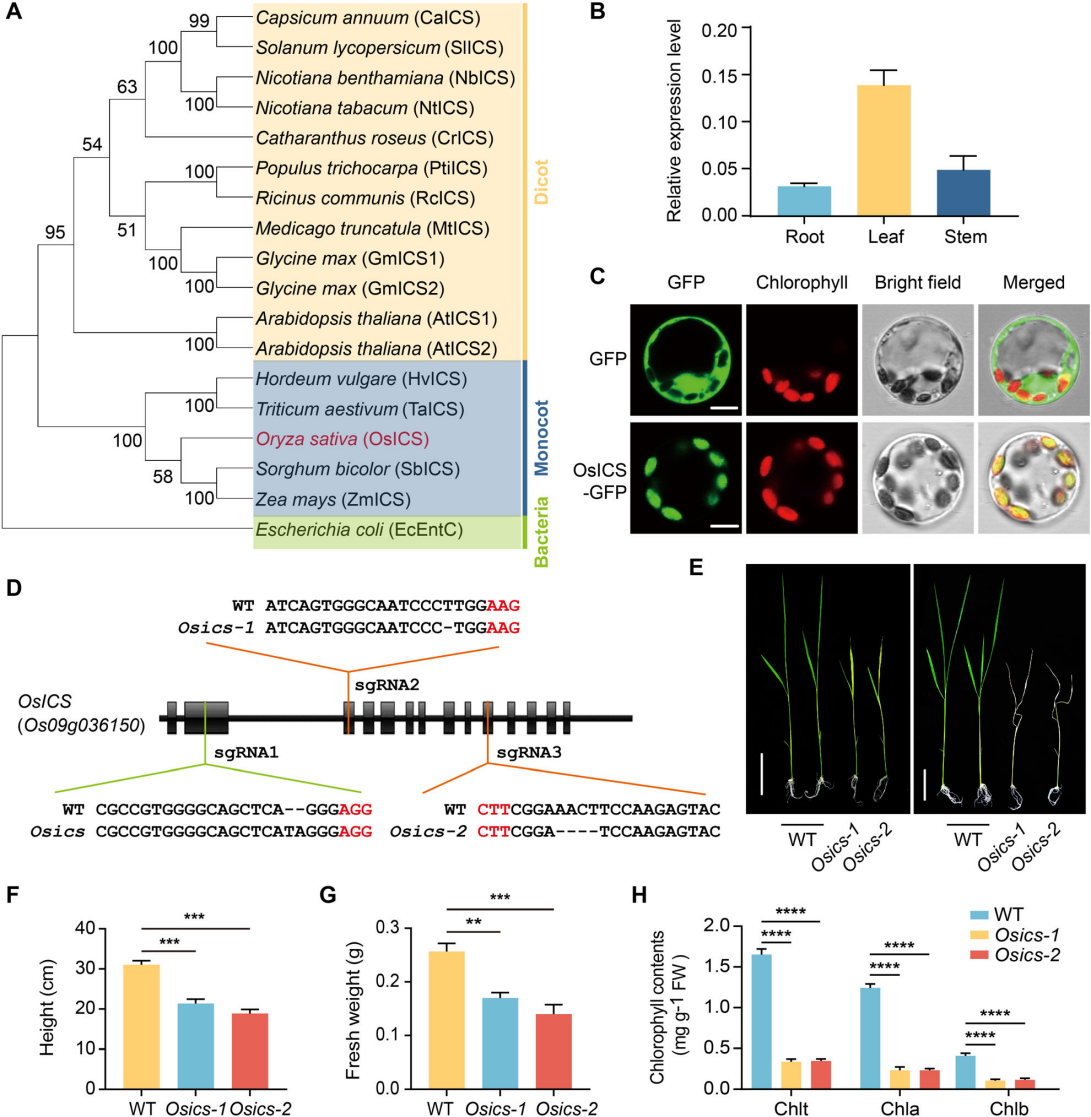
*OsICS* knockout mutants exhibit severe growth defects. **A** Phylogenetic analysis of ICS proteins from rice and other plant species. The OsICS is highlighted in red. EntC, *Escherichia coli* enterobactin-specific isochorismate synthase; ICS, isochorismate synthase. **B** The expression levels of *OsICS* in root, stem, and leaf of wild-type (WT) plants at the seedling stage. Data are represented as mean ± SD of three biological replicates. **C** Subcellular localization of the OsICS protein in rice protoplasts. **D** *OsICS* knockout mutants. sgRNAs with NGG and NAG are designed as PAMs. The PAM sequences are indicated in red. The black line represents the genome sequence, and black boxes represent exons. Sequences of the sgRNA-induced *OsICS* mutations in the individual mutant lines are shown. **E** Phenotypes of 15-day-old (left) and 20-day-old (right) seedlings of the wild-type plants and *Osics* mutants. Scale bar, 5 cm. **F**, **G** Plant height (**F**) and fresh weight (**G**) of the 15-day-old wild-type and *Osics* seedlings. **H** Chlorophyll contents, including total chlorophyll (Chlt), chlorophyll a (Chla), and chlorophyll b (Chlb) of leaves from the 15-day-old wild-type and *Osics* seedlings. *FW* fresh weight. Data are represented as mean ± SD of three biological replicates. **P < 0.01, ***P < 0.001, ****P < 0.0001 (two-tailed Student’s *t* test)

Unlike *Arabidopsis*, which harbors two *ICS* homologs, the rice *Nipponbare* genome contains a single-copy gene of *ICS* (*Os09g036150*). A maximum likelihood (ML) phylogenetic tree was constructed using the deduced amino acid sequences of OsICS and sequences of ICS proteins from other plant species, and OsICS clearly clusters together with other monocot ICSs (Fig. 1A). *OsICS* is expressed in the root, stem, and leaf of rice, with the highest expression level observed in the leaf (Fig. 1B). Analysis of the OsICS using the online tool (https://predictprotein.org/) predicted a chloroplastic localization. To confirm the subcellular localization of OsICS, *OsICS* was fused in frame with *green fluorescent protein* (*GFP*) and then transiently expressed in rice protoplasts. Confocal microscopy imaging of the transformed cells revealed that OsICS-GFP fluorescence colocalized with chlorophyll fluorescence (Fig. 1C), supporting that the OsICS protein is localized in the chloroplast, where the chorismate substrate is present.

### OsICS knockout mutants exhibit severe growth defects

We used the CRISPR/Cas9 system to generate rice mutants of *OsICS*. Initially, we designed a single-guide RNA (sgRNA1) targeting the second exon of *OsICS* with a canonical NGG PAM (Fig. 1D). Due to the high editing efficiency of the sgRNA1, *Osics* mutants obtained were all homozygous and seedling lethal. Consequently, these homozygous rice *ics* mutant materials could not be reproduced for further study. Therefore, we redesigned sgRNAs with NAG PAM (Fig. 1D), facilitating the generation of heterozygous mutants (Meng et al. 2018). The sgRNAs, sgRNA2 and sgRNA3, targeting exon 3 and exon 10 of *OsICS*, resulted in two independent heterozygous lines with a 1 bp and 4 bp deletion, respectively (Fig. 1D). Homozygous *Osics* mutants (named as *Osics-1* and *Osics-2*) were segregated and utilized for subsequent analyses.

**Fig. 2:**
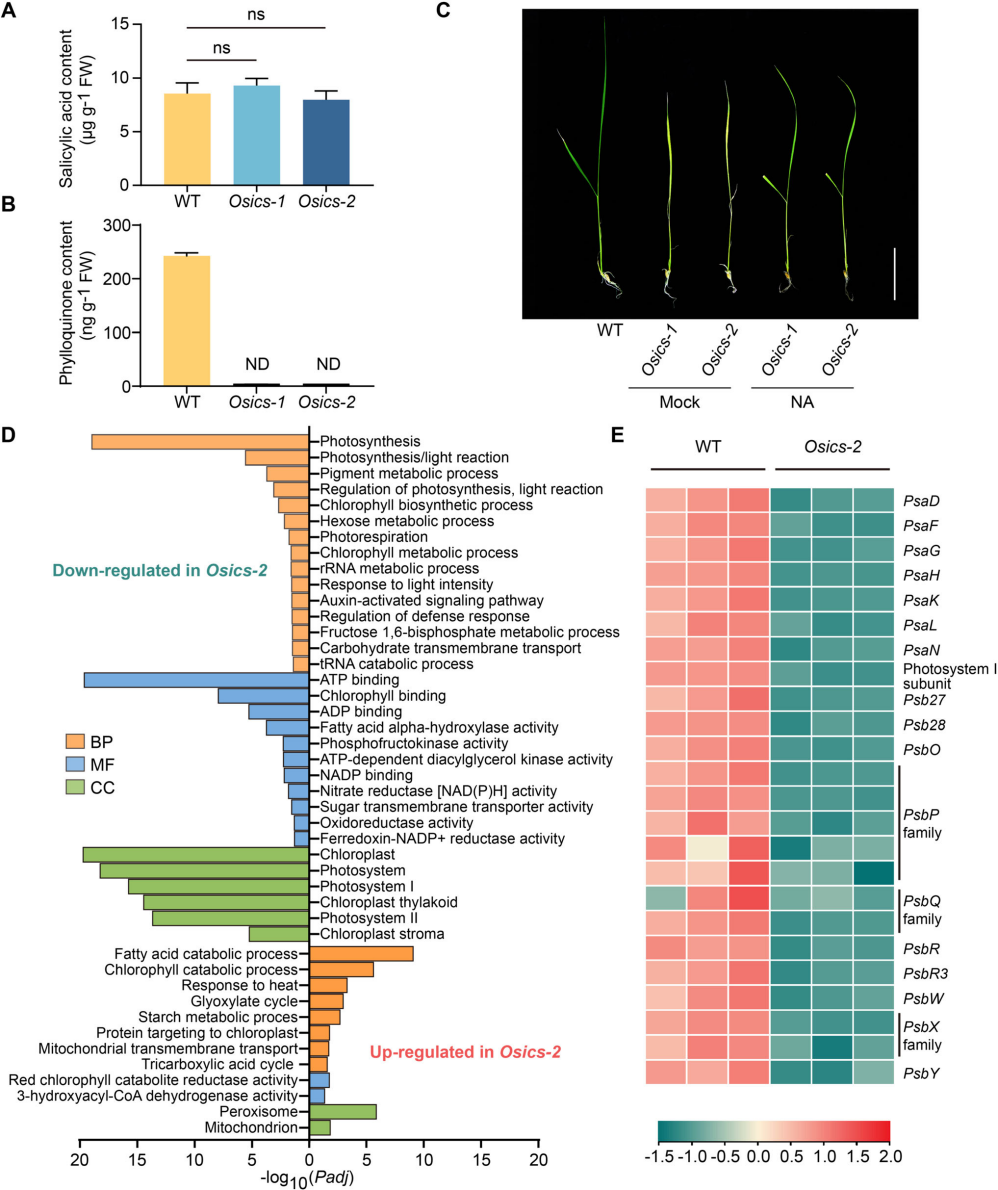
OsICS is required for phylloquinone, but not salicylic acid biosynthesis. **A**, **B** Contents of total SA (**A**) and phylloquinone (**B**) in 15-day-old wild-type plants and *Osics* mutants. Data are represented as mean ± SD of three biological replicates. ns indicates no statistical significance determined by two-tailed Student’s *t* test (*p* > 0.05). *ND* not detected. *FW* fresh weight. **C** Phenotypes of *Osics* mutants were rescued by exogenous application of 1,4-dihydroxy-2-naphthoic acid (NA). Rice plants were grown in Kimura B nutrient solution supplemented with either 0.1 mM NA or 0.1% dimethyl sulfoxide (mock) after seed germination. Scale bar, 5 cm. **D** Gene ontology enrichment analysis was performed on the differentially expressed genes (DEGs) in *Osics-2* compared with the wild-type plants. Bar charts showing the representative terms. *BP* biological process, *MF* molecular function, *CC* cellular component. **E** Heatmap showing the differential expression of genes encoding photosystem I and photosystem II related proteins. Gene expression levels are represented by a heatmap using the log_2_-transformed FPKM values. The accession number for the indicated genes are listed in Table S1

*Osics-1* and *Osics-2* both exhibited yellow leaves and dwarf at 15 days after germination (Fig. 1E, left). Consistent with their phenotypes, the accumulation of chlorophyll, as well as fresh weight and plant height of the two *Osics* mutants, were significantly lower than those of wild-type plants (Fig. 2F–H). Finally, the seedlings became wilted and died (Fig. 1E, right). These results support that *OsICS* plays crucial roles in rice growth and development.

### The Osics mutants are phylloquinone-deficient, but have unaltered basal SA levels

In addition to catalyzing the production of SA, ICS also plays a role in the biosynthesis of phylloquinone, another isochorismate-derived end product present in many plants. In the *Arabidopsis ics1ics2* double mutant, phylloquinone is entirely devoid, while retaining a low, but detectable residual level of SA (Garcion et al. 2008). To assess the content of phylloquinone and total SA in the homozygous *Osics* mutants, we conducted high performance liquid chromatography-tandem mass spectrometry (HPLC–MS/MS) analyses to precisely measure these two compounds. Both *Osics-1* and *Osics-2* mutants accumulated approximately 10 µg of total SA per gram of fresh weight, a level similar to that of wild-type plants (Fig. 2A). In contrast, phylloquinone was undetectable in *Osics-1* and *Osics-2* mutants (Fig. 2B). To confirm the involvement of ICS in phylloquinone biosynthesis, we exogenously supplied the mutant plants with 1,4-dihydroxy-2-naphthoic acid (NA), a precursor for phylloquinone. The *Osics* mutants were effectively rescued by NA, exhibiting green leaves and viable seedlings (Fig. 2C). These data indicate that the isochorismate synthesis is completely blocked in the *Osics* mutants and that basal SA is not derived from the ICS pathway in rice.

### Disruption of OsICS compromised the expression of photosynthesis-associated genes

To elucidate the potential impacts of disrupting ICS in rice, we carried out RNA-seq analysis of a mutant line (*Osics-2*) and wild-type plants to identify differentially expressed genes (DEGs) between them. Gene ontology (GO) enrichment of the DEGs showed that processes related to photosynthesis, photosynthesis/light reaction, and pigment metabolism within the biological processes (BP) category were highly enriched and down-regulated in the *Osics-2* mutant (Fig. 2D). Within the molecular function (MF) category, the majority of DEGs were associated with ATP binding and chlorophyll binding, which are crucial for light absorption and electron transfer during photosynthesis (Fig. 2D). Similarly, DEGs in the cellular components (CC) category were predominantly observed in chloroplast and photosystem (Fig. 2D). These data suggest that the process of photosynthesis is greatly affected in the *Osics-2* mutant. We further checked DEGs associated with photosystem I and photosystem II. The expression of genes encoding the major subunits of photosystem I (*PsaD*, *PsaF*, *PsaG*, *PsaH*, *PsaK*, *PsaL*, and *PsaN*) exhibited obvious down-regulation in the *Osics-2* mutant (Fig. 2E). Similarly, *PsbO*, *PsbP*, *PsbQ*, *PsbR*, *PsbR3*, *PsbW*, *PsbX*, *PsbY*, *Psb27*, and *Psb28* family genes of photosystem II were also down-regulated in the *Osics-2* mutant (Fig. 2E). Overall, our results demonstrate that disrupting *ICS* in rice results in compromised expression of photosynthesis-associated genes, supporting the function of ICS in phylloquinone biosynthesis.

## Discussion

Biosynthesis of SA is fundamental for understanding of its function and regulation. SA was originally proposed to be synthesized through the PAL pathway in plants, as indicated by studies based on radiolabeled compounds in cucumber plants (Meuwly et al. 1995). However, in *Arabidopsis*, a second pathway, known as the ICS pathway which is similar to that described in some *Pseudomonas* bacteria, was discovered (Wildermuth et al. 2001). The existence of the ICS pathway gained strong support from investigations involving *ics* mutants in *Arabidopsis*. In addition to its deficiency in pathogen-induced SA accumulation (Wildermuth et al. 2001), the *ics1ics2* double mutants exhibited a 80% decrease in total SA levels under basal conditions and a 95% decrease after UV induction in leaves (Garcion et al. 2008), which underscores the significance of the ICS pathway in biosynthesis of both basal and UV-induced SA in *Arabidopsis*. The indispensability of the ICS enzyme for SA production in response to biotic and abiotic stresses has been demonstrated in *Nicotiana benthamiana*, providing further evidence for the critical role of the ICS pathway in SA biosynthesis (Catinot et al. 2008; Shibata et al. 2010). It was also demonstrated in several other plant species that SA is primarily derived from the ICS pathway (Garcion et al. 2008). In this study, we successfully identified a functional *ICS* gene in rice and generated *Osics* mutants. These *Osics* mutants exhibited a yellowish leaf phenotype and were lethal at the seedling stage (Fig. 1), resembling the phylloquinone-deficient mutants *pha*, *aae14*, and *abc4* in *Arabidopsis* (Gross et al. 2006; Kim et al. 2008; Shimada et al. 2005). Importantly, no detectable phylloquinone was observed in the *Osics* mutants (Fig. 2), indicating that this ICS protein is solely responsible for the isochorismate production in rice. We measured the total SA content in these *Osics* mutants and found that the total SA content remained unchanged (Fig. 2). This observation provides genetic evidence for the existence of an ICS-independent pathway for SA production in rice. The biosynthesis of SA in rice appears similar to that in barley, in which SA is synthesized through an ICS-independent pathway as well (Qin et al. 2019). These observations provide evidence that the production of SA exclusively via an ICS-independent route is likely widespread in monocots. However, the pathway through which SA is synthesized in rice still remains for further investigation, with the PAL pathway being the most likely candidate.

It has been reported that some plants predominantly employ the PAL pathway for SA biosynthesis. For example, in the *PAL1*-silenced plants of *Aegilops variabilis*, basal SA levels in the roots decreased by 75%. In contrast, the SA levels were not changed in the *AevICS*-silenced plants (Zhang et al. 2021), suggesting that SA is primarily synthesized through the PAL pathway in *Ae. Variabilis*. In the case of rice, which has very high basal SA levels, its SA biosynthesis has been previously presumed to be through a PAL-dependent pathway (Xu et al. 2023). Nine genes have been annotated as *PALs* in rice, with *PAL1-7* co-localizing with disease resistance quantitative trait loci (QTLs), indicating their involvement in plant defense (Tonnessen et al. 2015). Among these, PAL6 has been shown to play a role in SA accumulation. The rice *PAL6* T-DNA insertion line exhibited great reduction in PAL activities and a decrease in SA levels (Duan et al. 2014). In addition, SA accumulation in rice was shown to be dependent on OsAIM1, a β-oxidase responsible for the production of benzoic acid (BA), which may serve as a precursor of SA in the PAL pathway. Knocking out *OsAIM1* leads to decreased SA levels (Xu et al. 2017, 2023). These observations underscore the significance of the PAL pathway for SA biosynthesis in rice. However, the regulation of SA biosynthesis in rice may differ from other plant species due to the high basal SA levels. Further research should be extended on identification of key SA biosynthesis enzymes in rice and on better understanding the multifaceted functions of SA in this major staple crop.

## Materials and methods

### Plant materials and growth conditions

Rice (*Oryza sativa* ssp. *japonica* cv. *Nipponbare*) seeds were germinated at 37 °C under dark conditions. After germination, rice plants were grown in Kimura B nutrient solution (Coolaber, China) in a growth chamber with 85% relative humidity under 13 h/11 h light/dark cycle at 28 °C/24 °C day/night temperatures. For NA treatment experiment, 5-day-old *Osics* plants were supplied with 0.1 mM NA or buffer (0.1% dimethyl sulfoxide) and the nutrient solution was renewed every 3 days.

### Phylogenetic analysis of ICS

Multiple sequence alignments of ICS proteins from different species were performed using ClustalW with default parameters. The maximum likelihood (ML) phylogenetic tree was constructed using MEGA 11.0. Accession number of ICS proteins are listed in Table S2.

### CRISPR/Cas9 editing vector construction and rice genetic transformation

The CRISPR v2.0 (http://crispr.hzau.edu.cn/CRISPR2/) was used for designing sgRNA. A pair of complimentary oligos corresponding to each sgRNA were synthesized commercially, and then were annealed and inserted at the *Bsa*I site of the vector pHUE411 (Xing et al. 2014). The construct was then introduced into rice embryogenic calli via *Agrobacterium tumefaciens* as described (Hiei et al. 1994). The edited plants were identified with PCR. The primers used are listed in Table S3.

### Chlorophyll, SA and phylloquinone measurements

Chlorophyll content was determined as described, with minor modifications (Alam et al. 2022; Li et al. 2022). Fresh leaves (approximately 30 mg) were ground to powder with liquid nitrogen and extracted with 1.8 mL of 80% acetone. After centrifuged at 10,000 rpm for 5 min, the resulting supernatants were measured with spectrophotometric scanning at 663 nm and 645 nm for chlorophyll a (Chla) and chlorophyll b (Chlb), respectively. For SA and phylloquinone measurements, the leaves (approximately 0.1 g) of 15-day-old rice seedlings were collected and ground into powder in liquid nitrogen. Extraction and measurement of SA and phylloquinone were performed as previously described (Glauser et al. 2014; Lohmann et al. 2006).

### Subcellular localization of OsICS

OsICS was fused in frame with GFP by cloning the coding sequence without the stop codon of *OsICS* into the pJIT163-ubi-GFP vector (Wang et al. 2014). The pJIT163-Ubi-GFP (control) and pJIT163-Ubi-OsICS-GFP constructs were transformed into rice protoplasts as described (Shan et al. 2014). The fluorescence images were obtained with a Leica TCS SP8 laser scanning confocal microscope.

### RNA-seq and quantitative RT-PCR assays

Total RNA was extracted using a RiboPure kit (Invitrogen, USA). RNA-seq was conducted using the Illumina NovaSeq platform, and DEGs were detected by edgeR with a threshold absolute value of |log_2_(fold change)|≥ 1 and *Padj* (adjusted *P*-value) ≤ 0.05 (Robinson et al. 2010). GO enrichment analysis of DEGs was performed using Gene Ontologies (https://www.geneontology.org/). For quantitative RT-PCR (qRT-PCR), 1 µg of total RNA was reverse-transcribed into first-strand cDNA using Superscript Reverse Transcriptase III (Invitrogen, USA). qRT-PCR was performed using the SYBR Premix Ex Taq™ kit (Takara, Japan). *Ubiqutin* was used as the internal control. Normalized expression levels were determined using the 2^−ΔCt^ method. The primers are listed in Table S3.

## Supplementary Information

Below is the link to the electronic supplementary material.

Supplementary file1 (PDF 131 KB)

## Data Availability

All data generated in this study are available in the paper.
