# Supplementary material for: Isochorismate synthase is required for phylloquinone, but not salicylic acid biosynthesis in rice
Source: aBIOTECH. 2024 May 24;5(4):488–96. doi: 10.1007/s42994-024-00166-4 (PMC11624176; doi:10.1007/s42994-024-00166-4)
Supplement: Supplementary file 1 — Supplementary file1 (PDF 131 KB) [file 42994_2024_166_MOESM1_ESM.pdf]

SUPPORTING INFORMATION

Isochorismate synthase is required for phylloquinone, but not salicylic acid biosynthesis in rice

Zengqian Wang[1,2,§], Guiqing Yang[1,2,§], Dandan Zhang[2], Guangxin Li[1], Jin-Long Qiu[2,#], Jie Wu[2,#]

[1] College of Agriculture, Shanxi Agricultural University, Jinzhong 030801, China

[2] State Key Laboratory of Plant Genomics, Institute of Microbiology, Chinese Academy of Sciences, Beijing 100101, China

[§] These two authors contribute equally to this work.

[#] To whom correspondence should be addressed. E-mail: qiujl@im.ac.cn; wujie1987519@163.com

Table S1. Accession number of rice genes described in this study.

Gene	Accession Number
Ubiquitin	*Os03g0234200*
ICS	*Os09g036150*
PsaD	*Os08g0560900*
PsaF	*Os03g0778100*
PsaH	*Os05g0560000*
PsaG	*Os09g0481200*
PsaK	*Os07g0148900*
PsaL	*Os12g0420400*
PsaN	*Os12g0189400*
photosystem I reaction centre subunit N family gene	*Os03g0731100*
PsbO	*Os01g0501800*
PsbP family genes	*Os01g0934400*
PsbP family genes	*Os07g0141400*
PsbP family genes	*Os07g0275100*
PsbP family genes	*Os08g0504500*
PsbP family genes	*Os12g0564400*
PsbQ family genes	*Os02g0631100*
PsbQ family genes	*Os07g0544800*
PsbR	*Os07g0147500*
PsbR3	*Os08g0200300*
PsbW	*Os01g0773700*
PsbX family genes	*Os03g0343900*
PsbX family genes	*Os07g0673550*
PsbY	*Os08g0119800*
Psb27	*Os03g0333400*
Psb28	*Os01g0938100*

Table S2. Accession number of ICSs used for the phylogenetic analysis.

Species	Accession Number
Oryza sativa	NP_001390677
Arabidopsis thaliana	NP_565090, ACC60228
Catharanthus roseus	CAA06837
Populus trichocarpa	ACX46383
Medicago truncatula	AES89520
Glycine max	KAH1256887, KAH1265858
Ricinus communis	EEF52128
Capsicum annuum	AAW66457
Solanum lycopersicum	ABJ98719
Sorghum bicolor	XP_021308739
Zea mays	NP_001143210
Nicotiana benthamiana	BBA45710

Nicotiana tabacum	BBA45709
Hordeum vulgare	KAE8803166
Triticum aestivum	NP_001392901
Escherichia coli	AAA16100

Table S3. Primers used in this study.

**Primer Name**	**Sequence (5'-3')**
**For genotype identification**	
sgRNA1-F	CCCGTCGGCGAGACTCGTACGTAC
sgRNA1-R	GGCTCGGTCCCGCGGAAGAACAC
sgRNA2-F	TGGTCTTGGCCCATACTGTGTC
sgRNA2-R	ACATGACTGACAGAATTGGAAGAAAGAG
sgRNA3-F	TCTCCAGATGATTTAAGGCGATATCAAC
sgRNA3-R	TGGGCTCGGATGAAGAGTATTTAGG
**For qRT-PCR Analysis**	
ICS-F	AGCATAAAGAAGAAGCTGGAGATGATC
ICS-R	ACAGGTGTTGTACTCTTGGAAGTTTC
